# The Comparative Analysis of the Coatings Deposited on the Automotive Parts by the Cataphoresis Method

**DOI:** 10.3390/ma14206155

**Published:** 2021-10-17

**Authors:** Wojciech Skotnicki, Dariusz Jędrzejczyk

**Affiliations:** Department of Mechanical Engineering Fundamentals, University of Bielsko-Biala, Willowa 2, 43-309 Bielsko-Biala, Poland; djedrzejczyk@ath.bielsko.pl

**Keywords:** paint coating, corrosion resistance, surface preparation, automotive

## Abstract

The paper presents preliminary research focused on the determination of the influence of surface preparation on the quality of the paint coating obtained by the cataphoresis method (KTL). The tests were carried out on steel parts used in the construction of trailers and truck bodies. The first research group consisted of cold-rolled and chemically cleaned parts, the second group were mechanically cleaned with abrasive blasting. In order to determine the influence of surface treatment on the corrosion resistance of the tested coatings, besides a corrosion test, roughness measurements were also carried out. Tests were performed on the crude surface and after coating deposition. Moreover, tests were supplemented by measuring the thickness of the coating using the magnetic induction method and the hardness with the use micro and nano hardness testers. Measurements of the tribological parameters under dry friction conditions were performed using a T11 tester. The corrosion resistance of the applied coatings was determined in a salt spray test. The obtained results were compared to those that were determined for different zinc coatings. It has been shown that the method of base steel surface preparation affects every measured parameter and property of tested paint coatings. The quality of the coating deposited on the steel base after chemical cleaning is much better than the one applied to the sandblasted surface. The measured corrosion resistance of the tested paint coatings is only greater than the corrosion resistance of the lamellar zinc coating. The other zinc coatings (galvanic, hot-dip, sherardized) show corrosion resistance by an order of magnitude higher.

## 1. Introduction

Despite the constant development of anticorrosion systems, the data from NACE International shows that the effects of corrosion can account for 3.5 percent to 5.2 percent of global gross domestic product [1]. In the largest car market in 2020 (Chinese market) [2], the annual direct corrosion costs amounted to 187.25 billion RMB (about $28 billion), representing 2.82% of the total value of the automotive industry [3]. A similar situation has been observed for a long time—for example, only the annual cost of corrosion in the motor vehicles industry of the USA (already in 2002) was estimated to be $23.4 billion in total [4]. The mentioned value includes: increased manufacturing costs due to corrosion engineering and the use of corrosion-resistant materials ($2.56 billion); repairs and maintenance necessitated by corrosion ($6.45 billion) and corrosion-related depreciation of vehicles ($14.46 billion). The problems with material durability in natural and artificial environments are extremely important in the designing of the steel structures and the metal parts of machines. Corrosion destruction is one of the main sources of material loss, environmental pollution and a number of air, road and sea disasters [5,6,7].

Structural elements are made of a wide range of materials that require various types of corrosion protection. Parts applied in the automotive industry (profiles, bolts, draw-pieces) are produced from different metallic materials, ranging from common carbon steel, alloy steel, stainless or corrosion resistant steel to aluminum, magnesium and titanium alloys [8,9]. Many mechanisms have been investigated and applied to prevent or protect metals from corrosion, such as barrier [10,11,12], inhibitors [13], and cathodic protection [14,15]. In order to improve the lifetime of the vehicles, many types of coated steels, chemical conversion treatments and paint systems were developed, together with optimization of automobile design, and also additional treatments such as sealers and waxes, were applied [16]. Pressure to reduce the production results in more and more often structural elements made from cheaper materials, which guarantee only the appropriate mechanical properties—the anticorrosion properties are improved by additional treatment—for the coating application. Moreover, the corrosion-resistant coatings reveal two basic advantages: simplicity and efficiency [12]. Although smart materials with self-healing properties are already used as coatings [17], and this effect can also be achieved using shape memory polymers [18], the environmental and economic factor seems to favor the traditionally used metals and film coatings.

Zinc is one of the cheapest elements among the traditionally used ones in the production of anticorrosion coatings (Zn, Cu, Ni, Cr) [19], and in addition, processes of Zn coating deposition are very simply—they do not require large financial outlays [20]. According to the report of the International Lead and Zinc Study Group [21], the global zinc production in 2016–2020 amounted to about 12 million tons yearly and more than half of the resources were used to protect the steel from corrosion in galvanic processes. Generally, zinc coatings applied to different elements are obtained by four methods: hot-dip galvanizing, electro-galvanizing, zinc lamella and sherardizing (thermal diffusion) [22,23,24].

Unfortunately, there are significant discrepancies in the literature data on the corrosion resistance of different coatings [25,26,27,28,29]. On the one hand, the accelerated comparative tests of the hot-dip (HD) and thermo-diffusion coatings (TD) carried out in a neutral salt spray environment [25] showed that the lower corrosion effect was in the case of a thermo-diffusion coating (1000 h to iron corrosion; for the hot-dip zinc coating—250 h to iron corrosion). Next, in the paper [26], it was stated that thermo-diffusion protection improves corrosion resistance of the high-strength steel bridge cable wire, but the higher corrosion resistance and the lifetime longer than conventional hot-dip-galvanized treatment can be achieved under special circumstances. In the paper [27], the thermo-diffusion protected steel sheet was less resistant to corrosion than the hot-dip zinc galvanized material. Other data [28] reports that the galvanic-coated bolts show the least corrosion resistance in salt spray environments compared to hot-dip and thermo-diffusion coating. After 1500 h in a salt spray environment, the bolts with galvanic protection were completely corroded, while on the hot-dip coating, the galvanized bolts, mainly the nut and parts of the thread, were corroded, and the thermo-diffusion galvanized bolts had only traces of iron corrosion. Generally, in the literature there is an opinion that both mechanical and anticorrosive properties of zinc diffusion layers are far exceeded compared with those of other zinc coatings without any diffusion interaction with metal base [29].

Kalendova [30] claims that corrosion resistance of lamella coatings depends on the size of zinc flakes—the smaller the flakes, the higher the anti-corrosion properties of the coating. According to Chinese researchers [31], the resistance of lamella coatings depends on the content of epoxy resin inside the layer. The highest corrosion protection values are achieved in the range of 30–35% of resin. For comparison, corrosion resistance of the cataphoretic coating in salt spray is estimated in the range 480–1000 h [32]. Research conducted in the article [16] have shown that the corrosion rate of vehicles (zinc coated elements) depends on climate conditions—severe corrosion was stated in Europe, while corrosion in Thailand was milder than that in the snowy regions, because of higher temperature and relative humidity in Southeast Asia.

In the automotive industry, cataphoretic coatings have frequently been used since the 1970s to avoid corrosion on suspension components [33]. The advantages of the process include, among others, the possibility of easy automation of the entire painting process; the differences in the thickness of the coating are small, even in hard-to-reach zones, which cannot be painted by powder application. Cataphoretic painting does not cause streaks, the surface coverage efficiency is very high, the losses are less than 10%, and the emission of volatile organic compounds VOC into the atmosphere is very limited. The cataphoretic coating ensures a similar corrosion resistance on the inner and outer surfaces, independent of the element geometry [33].

The inconvenience of cataphoretic varnishing is the need to introduce special drain holes in the painted structures, e.g., in the automotive industry, antennas on motor vehicles are not placed on roofs for better signal reception, but just to cover one of the drain holes [32]. However, the biggest enemy of the graphite-gray coating is UV rays. When tested according to EN ISO 16474-2 standard [34], the KTL coating loses its properties in less than 100 h. Suspension elements that are not exposed to sunlight are not subjected to further treatment [32].

The ability to correctly determine corrosion hazards is one of the most important elements necessary for the proper design of anti-corrosion protection systems. The PN-EN ISO 12944-2 [35] standard lists aggressive industrial environments, marine areas and the main arteries of large cities at rush hours, as the most endangered environments. The corrosion rate is influenced by several factors, such as: humidity, temperature and pollution (chemical composition). Various chemical compounds cause moisture water to become an electrolyte, conducive to the formation of corrosion centers [36,37]. Anti-corrosion protection of vehicles during severe winters is a particularly difficult issue; this problem attracted attention in the 1970s, since the salt is applied on roads to prevent accidents in snowy areas (USA, Europe and Japan) [16].

The effectiveness of anti-corrosion phenomena of the paint coating depends on the interaction of anti-corrosion pigments, the coating’s tightness and adhesion to the base surface [38]. Surface preparation before the coating deposition is the most important and difficult step in the corrosion protection technology [39,40,41,42,43]. Surface of steel may be contaminated by various media (the products of the reaction with the surrounding environment (scale, rust); fats, oils, lubricants, pollutants deposited from the atmosphere (dust and water-soluble salts, usually invisible to the human eye), the removal of which usually requires several operations.

Taking into account the above literature analysis, a similar cost of zinc and KTL coatings (depending on the time of protection and the size of the protected element [44,45]) and similar corrosion resistance of the compared coatings [25,26,27,28,29,30,31,32,33,34], in this paper, the research on the impact of the base (steel) surface preparation on the properties of KTL coating was investigated. The results obtained were compared to those presented in the authors’ earlier studies on zinc coatings [46,47,48].

## 2. Materials and Methods

**Figure 1:**
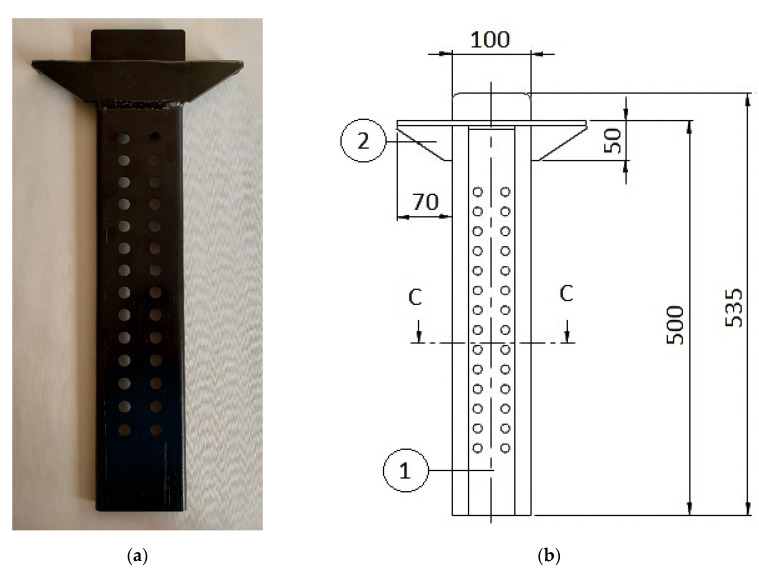
The steel guide rail view (**a**) and dimensions (**b**).

During the experiment, the properties of the coatings deposited on the steel guide rail (made of S355J2G3 steel, used in the truck semitrailer—Figure 1) were tested. The guide rail consists of two cold-rolled elements: guide plate (1) and bracket (2). The chemical composition (wt.%) of the applied structural steel S355J2G3 (typical material for truck frames and car bodies [49]) was as follows: 0.20% C, 1.55% Mn, 0.50% Si, 0.030% P, 0.032% S, 0.31% Cr, 0.07% Mo, 0.283% Ni (according to the producer’s certificate).

Samples for testing were divided into two groups (according to the guidelines of the manufacturer of steel guides) due to the method of surface preparation before applying the varnish coating. The first group (GROUP 1) included samples for which chemical treatment was applied. A 12% solution of hydrochloric acid was used to clean the surface. The temperature of the solution was in the range of 35–45 °C, and the etching time was 10 min. Samples in the second group (GROUP 2) were cleaned only mechanically—by abrasive blasting. Sandblasting of the samples was carried out using a PEKO-140 cabin air blast machine with a cylindrical nozzle made of tungsten carbide. The angle of inclination of the cleaning nozzle to the treated surface was 45° (operating pressure of 0.4 MPa). A95 alloxite with a grain size of 1–2 mm and a hardness of 1355 HV was used for sandblasting. The assessment of the surface cleanliness after abrasive blasting was carried out macroscopically, according to the PN-ISO 8501-1:2008 standard [50]. Cataphoretic painting involved immersing the cleaned component in a bath of Cathoprime QT82-9436 water-soluble paint (BASF Coating AG, Münster, Germany) and passing an electric current simultaneously. Cataphoretic painting was carried out in an acidic solution with a pH of 5.8–6.5. The thickness of the coating was adjusted by the change of values of the current voltage and electrolyte deposition time. A voltage range of 230–270 V was applied for 3 min. After painting, the paint coating was dried in 180 °C within 30 min.

The following parameters were analyzed during the investigations (on the crude and painted surface):

- the surface roughness (the contact profilometr—Perthometer Concept (MAHR) (Mahr GmbH, Göttingen, Germany) , with 3D software and a cone-shaped mapping blade (R = 2 μm, Θ = 90°);
- the coating thickness (PosiTector 6000 MP magnetic induction tester (DeFelsko, Ogdensburg, USA) with 90° depth finder–DeFelsko (DeFelsko, Ogdensburg, USA) and microscopic examinations in accordance with the PN-ISO 19840:2009 standard [51]);
- the hardness changes in the outer coating layer—measured in accordance with the standard PN-EN ISO 14577-1:2015 [52] (Anton Paar NHT2 tester (Anton Paar Germany GmbH, Ostfildern-Scharnhausen, Germany) with Berkovich indenter (Anton Paar Germany GmbH, Ostfildern-Scharnhausen, Germany) and max. load 20 mN);
- the hardness at the cross section of subsurface layer of steel (Vicker’s HV 0.02, Mitutoyo Micro-Vickers HM-210 A device 810–401 D (Mitutoyo, Kawasaki, Japan) );
- the friction coefficient (T11—pin on disc tester—the rotational speed of the sample was equal to 30 rpm. During the test the counter-sample made of 23 MnB4 steel and a diameter of 4 mm was used. The friction force and temperature (22–32 °C) at a unit load of 0.25 MPa were continuously recorded);
- the coating corrosion resistance (Ascott CC1000iP salt chamber—tests were carried out in accordance with PN-EN ISO 9227:2017-06 standard [53]. The parameters of accelerated corrosion tests in the salt chamber (NSS) were as follows: corrosive medium—NaCl 50 ± 5 g/dm^3^, density of the solution −1.035 g/cm^3^, fall value—1.033 g/cm^3^, solution’s pH—6.7, air pressure—1 bar, temperature inside the chamber—35 °C. After removal from the chamber, the test parts were cleaned in an aqueous 12% HCl solution with the addition of a 0.1% corrosion inhibitor);
- the microstructure of KTL coating structure and steel, with the use of an optical microscope (Axiovert 100 A (Carl Zeiss GmbH, Oberkochen, Germany) ) and EDS analysis (scanning electron microscope EVO 25 MA Zeiss (Carl Zeiss GmbH, Oberkochen, Germany) with an EDS attachment).

## 3. Results and Discussion

### 3.1. The coatings’ Topography and Geometry Measurements

During the investigation, the different coating profile parameters were measured using the contact profile measurement gauge—Perthometer Concept (MAHR) as well as optical PhaseView-ZeeScan system (version 2.7).

**Figure 2:**
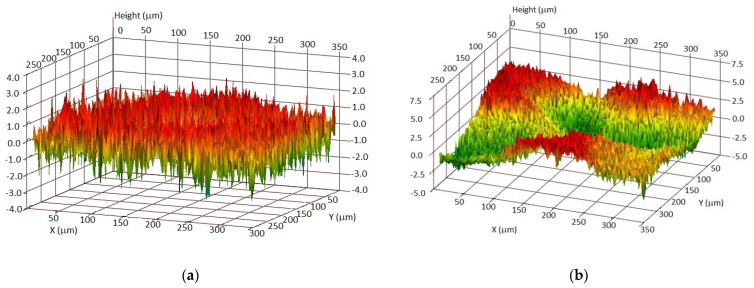
The topography of the tested crude surface before deposition of a varnish coating: after chemical cleaning (**a**) and after abrasive blasting (**b**).

Due to the applicative nature of the research, the analysis concerned only the most commonly compared parameter—Sa (arithmetic mean height). An example of the topography of crude surface after chemical cleaning and after abrasive blasting is presented in Figure 2.

**Figure 3:**
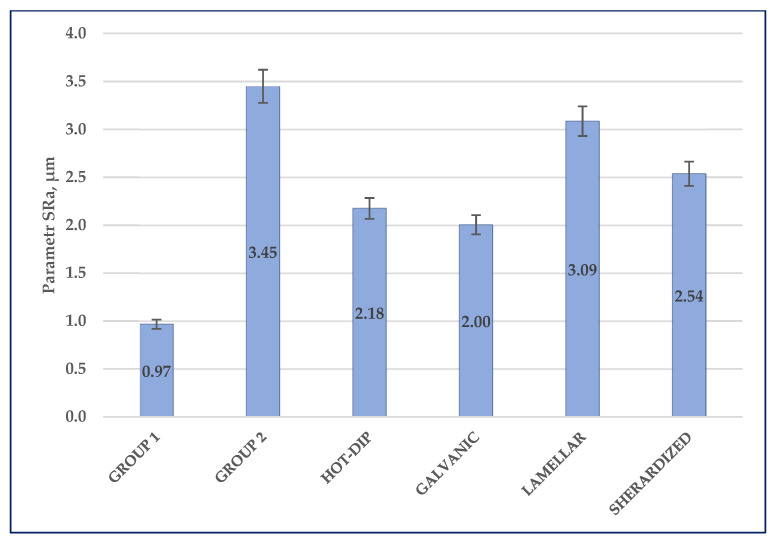
Results of surface topography measurement—SRa parameter.

The results of measurements of mean deviations from the mean line SRa for tested surfaces with a coating are shown in Figure 3.

Measurements of the thickness of the applied varnish coatings were made for each group in ten randomly selected places/points. The results of the thickness measurements are presented in Table 1 (an arithmetic mean, standard deviation—s.d.).

The obtained results are compiled with the data on zinc coatings investigated in the previous authors’ experiments—Table 2 [48,54,55,56].

Thickness measurements confirm that the coating applied to the chemically cleaned surface was uniform (average thickness was 33 μm (s.d. = 1.62 (G1)). In the second case, the varnish coating was much more diverse. The minimum measured coating thickness was 18 μm, while the maximum value was 34 μm (s.d. = 6.03 (G2)). The average thickness of the varnish coating obtained on the mechanically cleaned surface was 26 μm.

The literature data [40] suggest that the surface roughness before application of the protective coatings may affect their performance. It is reported that too much roughness can cause a decrease in corrosion resistance and mechanical properties. The crude surface after sandblasting was less homogeneous and more developed than the chemically treated one. These differences were reflected in the measured roughness values: the surface roughness after sand blasting was SRa = 4.18 μm and was more than three times higher than the roughness of the chemically cleaned surface—SRa = 1.15 μm. After deposition of the varnish coating, the roughness decreased slightly (respectively to 0.97 (G1) and 3.45 (G2) μm). It is likely that the coating’s material fills smaller irregularities caused by the crude surface treatment. This suggestion was also confirmed by microscopic observations (Section 3.2).

The obtained paint coatings are closest to zinc galvanic and sherardized coatings in terms of thickness. The thickness of the hot-dip zinc coating is several times greater, while the lamella coating is twice as thin. The smallest roughness is shown by the varnish coating applied to the steel surfaces after chemical cleaning. The surface preparation by sandblasting results in a surface roughness that exceeds the roughness of zinc coatings.

### 3.2. Metallographic Observations and Micro-Hardness Distribution

**Figure 4:**
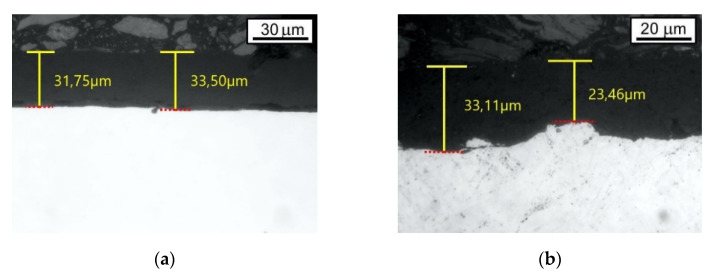
The microstructure of the analyzed coatings observed under the optical microscope: (**a**) Group 1, (**b**) Group 2.

**Figure 5:**
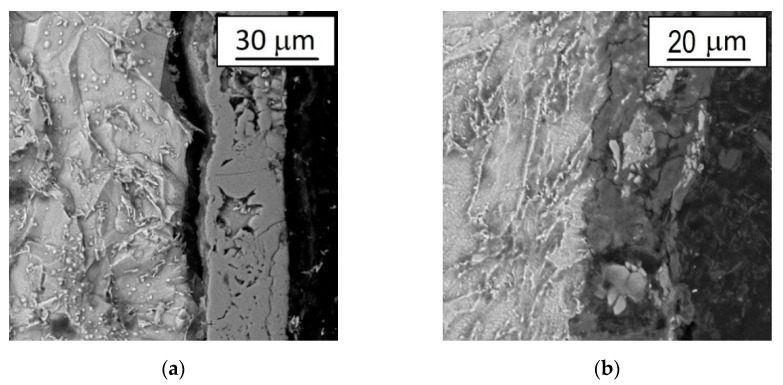
The microstructure of the analyzed coatings observed under the scanning microscope: (**a**) Group 1, (**b**) Group 2.

The microstructure of investigated coating is presented in Figure 4 and Figure 5. The observation with the use of the optical microscope (Axiovert 100 A) does not allow the structure of the applied coatings to be assessed. The image shown in Figure 5 is too contrasting, and the reflection of light rays from the varnish coating is too diffuse. Nevertheless, the observations made it possible to verify the thickness and uniformity of the studied coatings.

Data from Figure 4 are fully consistent with the information in Table 1, i.e., coatings deposited on the samples in Group 1 are more uniform and regular. Moreover, the observation with the application of scanning microscope (Figure 5) enabled the assessment of the microstructure at the cross section of the deposited coatings. Moreover, in this case, the “Group 1” coatings look much better: the structure is more compact, less cracked and less porous.

**Figure 6:**
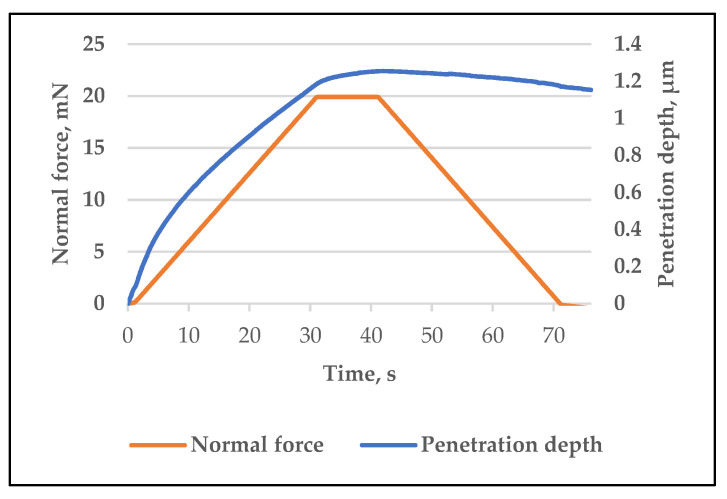
Graph registered during the hardness measurement of the varnish coating.

Measurements of the hardness of the applied varnish coatings were made for each group in ten randomly selected places/points (Figure 6). The results of hardness measurements, characterized by the depth of penetration of the indenter, and their arithmetic mean are presented in Table 3.

The lifetime of the coating depends on its properties: adhesion, hardness, flexibility, abrasion resistance, UV resistance and impact resistance [57]. The hardness measurement was aimed at determining the functional properties of the varnish coating depending on the type of surface on which they were applied. These studies did not show any significant relationships between the method of base surface preparation and measured hardness values. The average depth of penetration of the indenter for both groups was 1.2 ± 0.1 μm (which corresponds to the Vickers hardness value of 30 HV). Although the measured values are very similar, also in this case the standard deviation of the results of the first group was slightly smaller.

### 3.3. Friction Coefficient Measurements

**Figure 7:**
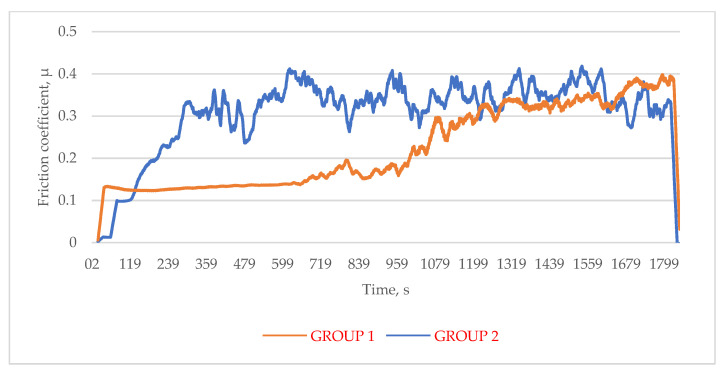
Change of the friction coefficient as a function of time.

The durability of the obtained coatings can be determined by their resistance to wear in conditions of dry friction. A counter-sample made of 23 MnB4 steel with a diameter of 4 mm was used during the research. Changes in the coefficient of friction as a function of time are shown graphically in Figure 7.

In the process of friction, two main stages can be distinguished. In the initial period of cooperation, the friction coefficient increases rapidly and after forming a contact, stabilizes. For a coating applied to a chemically cleaned steel base, three stages are visible in Figure 7. Creating a contact takes here much less time than in the case of the samples from Group 2 (G1—30 s; G2—300 s). Moreover, the value of the coefficient of friction achieved in the second stage is much lower (G1—0.13; G2—0.3). Only in the third stage (after approx. 1320 s) do the values of the coefficients for both groups reach a similar level (0.3).

**Figure 8:**
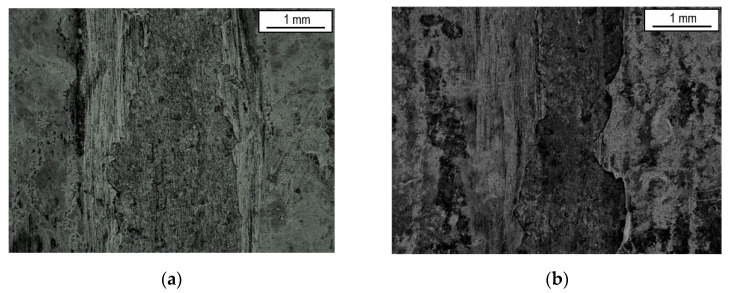
The macrostructure of the analyzed coatings after friction tests: (**a**) Group 1, (**b**) Group 2.

The more than doubling of the friction coefficient value (specimen G1) during the test (third stage) was due to significant damage to the coating during friction. In the case of the coating obtained on a mechanically cleaned surface (G2), after forming contact, the value of the friction coefficient has increased to over 0.3 in value and next varied over a wide range (0.24–0.41—the average coefficient of friction was 0.35). In addition, during the conducted research, uneven wear of the coatings was noticed, which was probably caused by the differences in their thickness and structure. The groove worn during testing was jagged and less regular for samples in Group 2—Figure 8.

The measured friction coefficients are compared to values measured for different zinc coatings in Table 4 [48,54]. It is obvious that a properly prepared base surface (G1) provides a value of the friction coefficient at the level of the lowest value measured for the zinc coatings (lamellar).

### 3.4. Corrosion Resistance Tests

**Figure 9:**
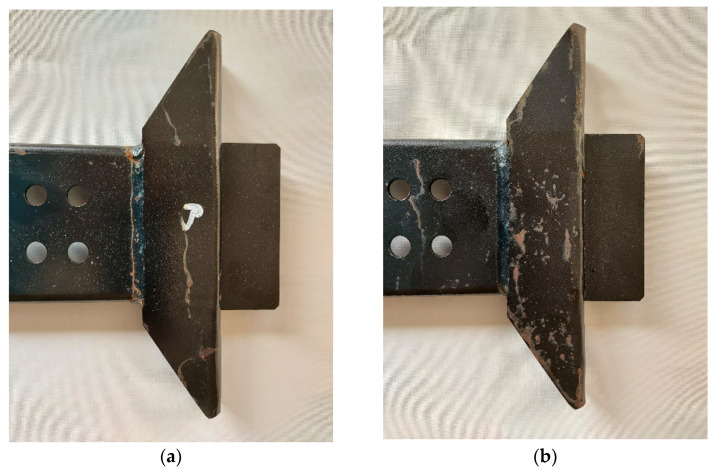
The view of the guide after corrosion resistance tests, (**a**) GROUP 1, (**b**) GROUP 2.

The final stage of the conducted research was a determination of the corrosion resistance of the tested parts in the salt chamber. The surface of the samples was assessed at intervals in accordance with the standard (24 h). The appearance of the guides after corrosion tests is shown in Figure 9.

The “red corrosion” appeared after 72 h on the surface of the tested parts from both groups. The research was continued and lasted 168 h. After this period, the corrosion test was stopped and the parts were checked macroscopically. It should be noted that on the abrasive cleaned surface (Group 2), the area affected by “red corrosion” was greater. It may be a consequence of the changes described in the previous points (differences in roughness, uniformity, microstructure, etc.) In addition, mechanical treatment can apply layers of dust and abrasive residues on the steel surface, which usually contribute to the development of corrosion.

In Table 5, the corrosion resistance of the coating parts protected by paint is compared to resistance of the four most popular zinc coatings determined in the same conditions. It is obvious that the measured corrosion resistance of the test parts is only better than the corrosion resistance of the lamellar zinc coating. The other zinc coatings show corrosion resistance by an order of magnitude higher. Therefore, zinc coatings with greater resistance (hot-dip or galvanized zinc coatings) should be used instead of paint coatings to protect parts of the vehicle chassis in areas exposed to difficult winter weather conditions.

## 4. Conclusions

(1) The method of the base steel surface preparation affects every measured parameter and properties of the tested paint coatings (surface topography, roughness, coatings’ thickness and evenness, microstructure, hardness diversification, friction coefficient and corrosion resistance).
(2) The quality of the coating deposited on a steel base after chemical cleaning is much better than the one applied to the sandblasted surface.
(3) The smallest roughness is shown by the varnish coating applied to the steel surfaces after chemical cleaning. Surface preparation with the use of sandblasting makes its roughness reach the value exceeding the roughness of zinc coatings.
(4) Coatings deposited on samples in Group 1 (chemical cleaned) are much more uniform and regular (the microstructure is more compact, less cracked and less porous).
(5) The friction coefficient measured with reference to the chemical cleaned samples (G1) is much lower than in the case of coating applied to the sandblasted surface (G2) (G1—0.13; G2—0.35).
(6) A properly prepared base surface (G1) provides a value of the paint coatings’ friction coefficient at the level of the lowest value measured for the zinc coatings (lamellar).
(7) There is no essential influence of the base steel surface preparation method on the tested paint coating corrosion resistance in an NSS test. Time to observe the “red corrosion” was the same, only the corroded area was greater on samples prepared by sandblasting.
(8) The zinc coatings with a higher corrosion resistance (hot-dip or galvanized zinc coatings) should be used, instead of paint coatings, to protect parts of the vehicle chassis in areas exposed to difficult winter weather conditions (salt roads protection).

## Figures and Tables

**Table 1:** The results of the thickness measurement of the varnish coating.

No. of Measurement and the Thickness of the Varnish Coating, µm.											
	1	2	3	4	5	6	7	8	9	10	Average (s.d)
GROUP 1	31 MIN	35 MAX	32	35	33	34	31	35	34	32	33 (1.62)
GROUP 2	18 MIN	25	19	31	33	21	34 MAX	25	31	21	26 (6.03)

**Table 2:** Comparison of the varnish and zinc average coating parameters.

Varnish		Zinc Coating			
**Average Coating Thickness, μm**					
GROUP 1	GROUP 2	HOT-DIP	GALVANIC	LAMELLAR	SHERARDIZED
33	26	88 [54]	19 [54]	11 [54]	45 [48]
**Average coating roughness, μm**					
GROUP 1	GROUP 2	HOT-DIP	GALVANIC	LAMELLAR	SHERARDIZED
0.97	3.45	2.18 [54]	2.0 [54]	3.09 [54]	2.54 [55,56]

**Table 3:** The results of the indenter depth penetration during the hardness measurement of the varnish coating.

No. of Measurement and Depth of Penetration of the Indenter, µm											
	1	2	3	4	5	6	7	8	9	10	Average (s.d)
GROUP 1	1.2	1.3 MAX	1.1 MIN	1.1	1.2	1.2	1.1	1.1	1.3	1.1	1.2 (0.08)
GROUP 2	1.3 MAX	1.3	1.1	1.0 MIN	1.1	1.1	1.3	1.0	1.2	1.1	1.2 (0.12)

**Table 4:** Comparison of the varnish and zinc coatings average friction coefficient.

Varnish		Zinc Coating			
Average Coating Friction Coefficient, μm					
GROUP 1	GROUP 2	HOT-DIP	GALVANIC	LAMELLAR	SHERARDIZED
0.13	0.35	0.25–0.29 [48,54]	0.20–0.21 [54]	0.12–0.13 [54]	0.20–0.38 [48]

**Table 5:** Comparison of the varnish and zinc coatings average corrosion resistance.

Varnish		Zinc Coating			
Average Corrosion Resistance, h					
GROUP 1	GROUP 2	HOT-DIP	GALVANIC	LAMELLAR	SHERARDIZED
78	78	816 [54,56]	> 1000 [54,56]	600 [54,56]	24 [54,56]

## Data Availability

Data is contained within the article.
